# Prohibitin-induced, obesity-associated insulin resistance and accompanying low-grade inflammation causes NASH and HCC

**DOI:** 10.1038/srep23608

**Published:** 2016-03-23

**Authors:** Sudharsana R. Ande, K. Hoa Nguyen, B. L. Grégoire Nyomba, Suresh Mishra

**Affiliations:** 1 Department of Internal Medicine, University of Manitoba, Winnipeg, Canada; 2 Department of Physiology & Pathophysiology, University of Manitoba, Winnipeg, Canada

## Abstract

Obesity increases the risk for nonalcoholic steatohepatitis (NASH) and hepatocarcinogenesis. However, the underlying mechanisms involved in the disease process remain unclear. Recently, we have developed a transgenic obese mouse model (Mito-Ob) by prohibitin mediated mitochondrial remodeling in adipocytes. The Mito-Ob mice develop obesity in a sex-neutral manner, but obesity-associated adipose inflammation and metabolic dysregulation in a male sex-specific manner. Here we report that with aging, the male Mito-Ob mice spontaneously develop obesity-linked NASH and hepatocellular carcinoma (HCC). In contrast, the female Mito-Ob mice maintained normal glucose and insulin levels and did not develop NASH and HCC. The anti-inflammatory peptide ghrelin was significantly upregulated in the female mice and down regulated in the male mice compared with respective control mice. In addition, a reduction in the markers of mitochondrial content and function was found in the liver of male Mito-Ob mice with NASH/HCC development. We found that ERK1/2 signaling was significantly upregulated whereas STAT3 signaling was significantly down regulated in the tumors from Mito-Ob mice. These data provide a proof-of-concept that the metabolic and inflammatory status of the adipose tissue and their interplay at the systemic and hepatic level play a central role in the pathogenesis of obesity-linked NASH and HCC.

Hepatocellular carcinoma (HCC), which starts in the hepatocytes, is the fifth most common cancer and a leading cause of cancer related death worldwide^1^. The pathogenesis of HCC has been mostly associated with cirrhosis due to chronic infection by hepatitis B virus and hepatitis C virus, as well as toxic injury from alcoholism^2^. While a substantial number of cases cannot be explained by these etiologies, HCC is increasingly diagnosed among obese individuals^3^. Obesity and obesity-related disorders such as non-alcoholic fatty liver disease (NAFLD), non-alcoholic steatohepatitis (NASH), insulin resistance and type 2 diabetes exhibit an increased risk for developing HCC^4^. For instance, in a large prospective cohort of the Cancer Prevention Study in North American subjects, the relative risk of dying from liver cancer among men with a BMI ≥35 kg/m^2^ was 4.5 times higher compared to a reference group with normal body weight^5^. Similarly, in a Swedish cohort study of men, the relative risk of HCC in individuals with a BMI ≥30 kg/m^2^ was 3.1 times higher than in normal weight controls^6^. Similar findings have been reported from other parts of the world^7^,^8^,^9^. Obesity increases male HCC risk by 4–8 fold^10^, and also increases HCC risk in viral hepatitis^11^. Furthermore, obesity associated tumors appear to be more aggressive, have an increased risk of recurrence, and result in higher mortality^12^,^13^.

Hepatic manifestations of obesity and metabolic syndrome are collectively termed NAFLD, which is commonly associated with insulin resistance and hyperinsulinemia^14^. Emerging evidence suggests that hepatic mitochondrial dysregulation has a role in the development of NAFLD^15^,^16^,^17^. For example, lipotoxicity-related hepatocyte apoptosis has been shown to involve mitochondrial permeability transition opening^15^. Similarly, oxysterols induced hepatocellular toxicity and NAFLD also involve mitochondrial impairment^16^. In contrast, enhancing mitochondrial function has been reported to reduce hepatic steatosis caused by diet-induced obesity (DIO)^17^. In general, NAFLD is apparently benign, but approximately 20% of all cases present as NASH showing hepatocellular injury and inflammation, with a risk of progression to cirrhosis and HCC^18^,^19^,^20^. The overall public health impact of an association between NAFLD, NASH and HCC remains substantial considering the high prevalence of obesity and related metabolic conditions worldwide. Emerging evidence suggests that NAFLD is associated with the development of non-cirrhotic HCC^21^,^22^,^23^,^24^. For example, an analysis of the SEER-Medicare database identified a total of 17,895 cases of HCC, of which 2,863 cases (16%) were due to biopsy-proven NAFLD without evidence for other etiologies^23^. Remarkably, a total of 1,031 cases (36%) of these NAFLD-associated HCC were diagnosed in non-cirrhotic livers and 18% of these cases developed in isolated fatty livers without steatohepatitis^23^. These evidences suggest that cirrhosis is not necessary for the development of HCC in obesity. Because of the epidemic proportions of obesity, there is an increasing probability that adverse metabolic conditions coexist with chronic liver disease, and obesity-associated abnormalities may enhance the effect of other established risk factors of HCC. Several studies support this notion such as alcoholic liver disease and chronic viral hepatitis associated HCC^24^,^25^. While NASH arising from fatty liver disease seems to be an underlying lesion for some HCC cases, the extent to which the presence or the severity of NASH explains obesity-related HCC remains unclear.

A number of molecular mechanisms have been linked to obesity and its associated abnormalities that may facilitate the development of HCC, such as adipose tissue inflammation, hepatic lipotoxicity, and insulin resistance^24^,^25^. These and other pathological events in obesity have complex interactions while their relative contribution to the development of HCC in various stages of NAFLD progression remains to be determined. Because of the continuous increase in obesity associated hepatic steatosis worldwide, there is an urgent need to better understand the underlying mechanisms involved in obesity-linked HCC development. However, one major obstacle for the mechanistic study is the lack of suitable animal models that spontaneously develop obesity associated NAFLD, NASH and HCC in a progressive manner. For example, the majority of the studies investigating the impact of obesity on transgene or carcinogen-induced HCC development or progression have been done using DIO rodent models^1^,^26^,^27^,^28^. A major disadvantage of the DIO model is that it is very difficult to distinguish the effects of the diet from overweight/obesity, and obesity from insulin resistance, which often coexist. For instance, Tsumura Suziki Obese Diabetes (TSOD) mice have been reported to spontaneously develop obesity, diabetes, fatty liver and HCC^29^. However, TSOD mice develop obesity due to hyperphagia that persist throughout the disease course from the development of diabetes to HCC^29^,^30^. In addition, TSOD mice develop diabetes by ~3 months of age and HCC much later in life (i.e. by 12 months), making it difficult to discern the effect of obesity from coexisting diabetes. Moreover, a number of liver specific transgenic mice have been reported that spontaneously develop HCC, with or without NASH^31^,^32^,^33^,^34^; however, they do not develop obesity prior to NASH and HCC. Also, the mechanism involved in the development and progression of carcinogen-induced HCC (an agent-specific HCC due to direct insult of liver) may not apply to obesity-linked HCC in humans, which develop progressively due to systemic changes in the body. Thus, suitable preclinical models that incorporate natural history estimates of disease progression are needed for a better understanding of the mechanisms of obesity-linked NASH and HCC. In addition, a suitable animal model is required for appropriate and meaningful intervention and preclinical studies.

Recently, we have developed a novel transgenic obese mouse model (Mito-Ob) by prohibitin (PHB, also known as PHB1) mediated mitochondrial remodeling in adipocytes, independent of diet^35^. The Mito-Ob mice develop obesity in a sex-neutral manner; however, they develop obesity associated low-grade inflammation, impaired glucose homeostasis, insulin resistance, and consequently NAFLD in a male sex-specific manner^35^. In considering the prevailing notion through which obesity may promote NASH and HCC development, we followed the consequence of NAFLD along with insulin resistance and chronic low-grade inflammation on hepatic phenotype in aging Mito-Ob mice. Here we report that the male Mito-Ob mice spontaneously develop obesity-linked NASH and HCC progressively, independent of diet.

## Results

### Mito-Ob mice display sex differences in adipose tissue structure and function, and in metabolic dysregulation

**Figure 1:**
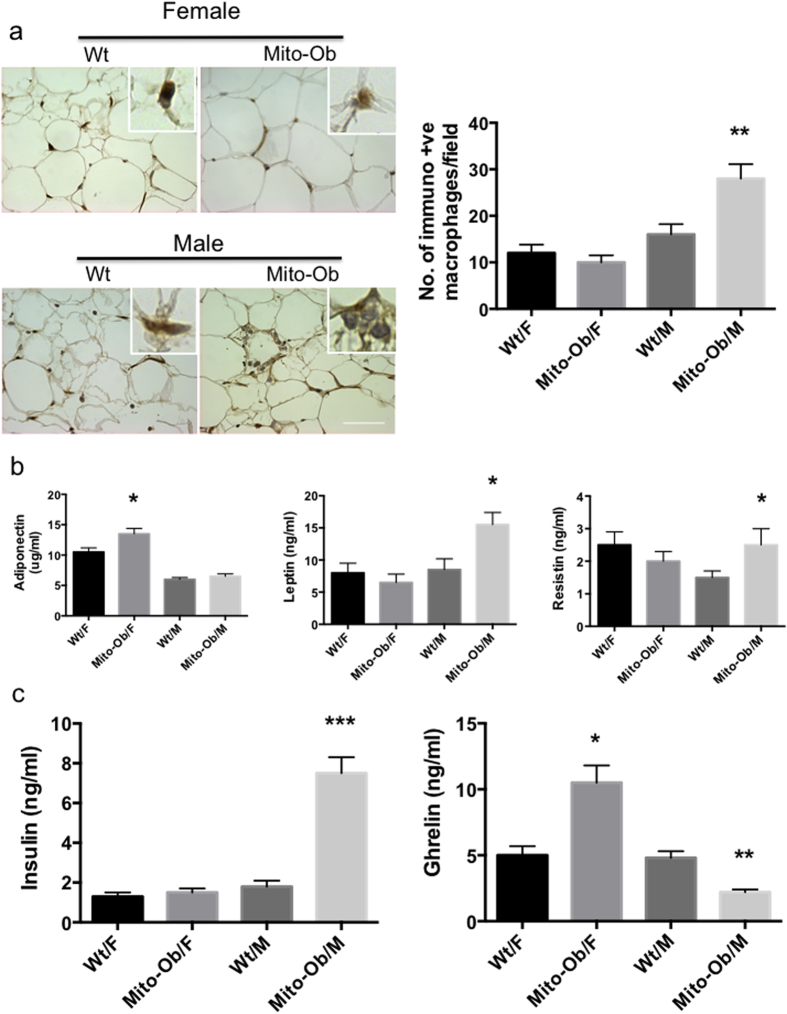
Mito-Ob mice display sex differences in adipose tissue structure and function, and in metabolic dysregulation. (**a**) Left panel: Representative photomicrographs showing immunohistochemical analysis of the inflammatory macrophages using anti-CD68 antibody in visceral adipose tissue from 9 months old Mito-Ob mice (Magnification 40X). Right panel: Histograms showing quantification of anti-CD68 positive macrophages as shown in the left panel. (n = 5–7 mice in each group). (**b**,**c**) Histograms showing serum adipokine and hormone levels in Mito-Ob mice at 9 months of age. Data are presented as mean ± SEM (n = 5–7 mice in each group). Two-way ANOVA (**a**–**c**) macrophage: P < 0.05 for genotype, P < 0.001 for sex, P < 0.01 for interaction; adiponectin: P = 0.17 for genotype, P < 0.001 for sex, P = 0.27 for interaction; leptin: P < 0.02 for genotype, P < 0.001 for sex, and P < 0.005 for interaction; Resistin: P = 0.72 for genotype, P = 0.72 for sex, and P = 0.30 for interaction; insulin: P < 0.0001for genotype, sex, and interaction; ghrelin: P < 0.05 for genotype, P < 0.0005 for sex and interaction. Asterisks indicate comparison between sex matched Mito-Ob vs Wt. *P < 0.05, **P < 0.01, ***P < 0.001 by Student’s t test. Wt – wild type; F – female; M – male.

Visceral adipose tissue inflammation plays a critical role in obesity associated systemic metabolic dysregulation in both humans and mice^36^. The sexually dimorphic metabolic phenotype of Mito-Ob mice^35^ prompted us to investigate the inflammatory status of their adipose tissues. Immunohistochemical analysis using macrophage specific marker anti-CD68 antibody (Abcam) revealed a significant increase in inflammatory macrophages in male Mito-Ob mice compared with wild-type mice (Fig. 1a). This difference was not observed between female Mito-Ob mice and control mice, despite comparable obesity and adipocyte hypertrophy in both the male and female Mito-Ob mice (Fig. 1a). Subsequent, data analysis by two-way ANOVA showed statistically significant interaction between sex and genotype (P < 0.01) (Fig. 1a). Consistent with the histological phenotype of adipose tissue, a sex-dimorphic change in adiponectin, leptin and resistin was also found. The adiponectin level was significantly upregulated in female Mito-Ob mice but not in male Mito-Ob mice in comparison with respective control mice (Fig. 1b). For adiponectin, no significant interaction was found between sex and genotype by two-way ANOVA (P < 0.27). Leptin and resistin levels were upregulated in male Mito-Ob but remained unchanged in female Mito-Ob mice in comparison with their respective control mice (Fig. 1b). Two-way ANOVA showed significant interaction between sex and genotype for leptin but not for resistin (Fig. 1b). Fasting serum insulin level was significantly upregulated in male Mito-Ob mice compared with control mice, whereas female Mito-Ob mice had insulin levels similar to wild type mice (Fig. 1c). Interestingly, Mito-Ob mice showed inverse alteration in the anti-inflammatory peptide ghrelin levels between males and females. Serum ghrelin level was significantly increased in female mice compared with wild type control mice (Fig. 1c). In contrast, male Mito-Ob had a significantly reduced ghrelin level compared with wild type control mice (Fig. 1c). Two-way ANOVA showed significant interaction between sex and genotype for insulin and ghrelin (Fig. 1c). This would imply that ghrelin might have a role in sexually dimorphic metabolic phenotypes in Mito-Ob mice. Taken together, these data suggest that Mito-Ob mice exhibit sex differences in visceral adipose tissue structure and function, and opposite changes in serum ghrelin levels that correlate with sexually dimorphic metabolic and inflammatory phenotypes of Mito-Ob mice.

**Figure 2:**
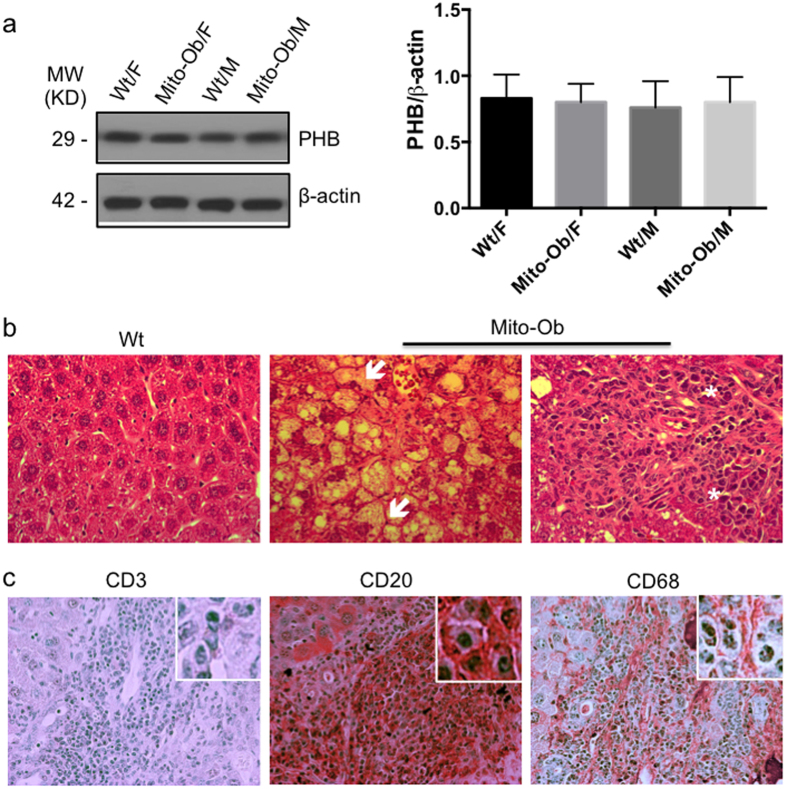
NAFLD in male Mito-Ob progresses to NASH with aging. (**a**) Left panel: Representative immunoblot showing PHB protein level in liver from 6 months old Mito-Ob mice in comparison with wild type mice. Beta-actin immunoblot is shown as loading control. Similar conditions were used for the electrophoretic analysis of proteins by Western immunoblotting. Right panel: Histograms showing quantification of PHB levels by densitometry as shown in the left panel. Data are presented as mean ± SEM (n = 3). No significant difference was found among groups. (**b**) Representative photomicrographs showing histological structure of the liver of male Mito-Ob mice and their age matched wild type littermates at 9 months of age. Hepatocyte ballooning and immune infiltration are indicated with white arrow and asterisk respectively. (**c**) Liver immunohisto-chemistry using macrophage and lymphocyte specific marker antibodies. (Magnification 40X; n = 4–6 mice in each group). MW – molecular weight; KD – kilo Dalton.

In our previous study, we have shown that Mito-Ob mice overexpress PHB in adipose tissue^35^ and in histiocytes/macrophages^37^. To confirm that there is no leaky overexpression of PHB from *aP2* promoter in the liver of Mito-Ob mice, we measured PHB protein levels by western immunoblotting. No significant difference in PHB protein level was found in the liver from wild type and transgenic mice suggesting that PHB is not overexpressed in the liver of Mito-Ob mice (Fig. 2a).

### NAFLD in male Mito-Ob mice progresses to NASH with aging

The sex specific hyperinsulinemia and ectopic fat accumulation in the liver of male Mito-Ob at 6 months of age, as found in our previous study^35^ led us to investigate the hepatic phenotype with aging. Histological examination of the liver at 9 months of age showed signs of NAFLD progression to NASH in male Mito-Ob mice as revealed by the characteristic ballooning of hepatocytes and lymphocyte infiltration (Fig. 2b). Increased inflammatory macrophages and lymphocytic infiltration in the liver was further confirmed by immunohistochemical analysis using macrophage and lymphocyte specific marker antibodies (Fig. 2c). Infiltrated cells were predominantly positive for CD20 and CD68, and only few were CD3 positive (Fig. 2c). Taken together, these data suggest that obesity associated NAFLD in male Mito-Ob mice spontaneously progress to NASH with aging.

### Male Mito-Ob mice spontaneously develop HCC with aging

**Figure 3:**
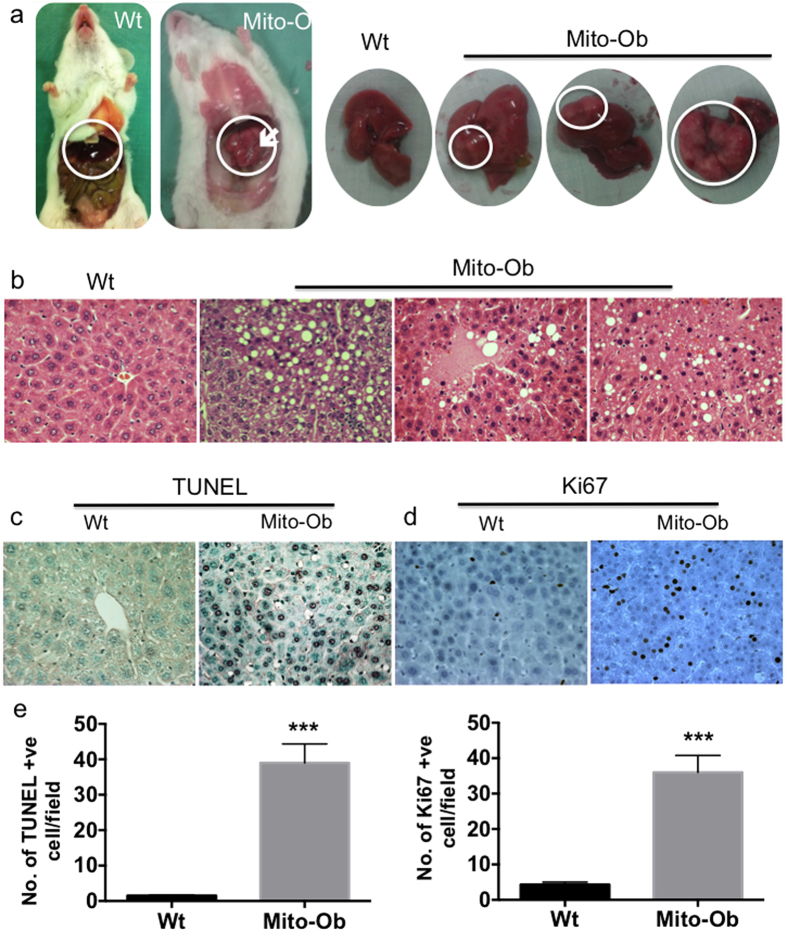
Male Mito-Ob mice spontaneously develop HCC with aging. (**a**) Representative photographs showing liver morphology from 12–14 months old Mito-Ob mice and their wild type littermates. Tumor in the male Mito-Ob mice is indicated with white arrow and circle. Representative photomicrographs showing histological architecture of H & E stained liver showing features of NAFLD, NASH and HCC (**b**), apoptotic cells death as determined by TUNEL assay (**c**) and cell proliferation as determined by anti-Ki67 antibody (**d**). Magnification 40X. (**e**) Histograms showing quantification of cell death and cell proliferation in the liver as determined in panel (**c**,**d**). Data are presented as mean ± SEM (n = 5–7 mice in each group). Asterisks indicate comparison between sex matched Mito-Ob vs Wt. ***P < 0.001 by Student’s t test. Wt – wild type.

Interestingly, in our follow-up study we found that male Mito-Ob mice start to develop pale to whitish nodular tumors (2–4 mm) on their liver surfaces around 10–12 months of age, which progressively grew into multifocal larger tumors (6–8 mm) by 12–14 months of age (Fig. 3a), with a prevalence of ~25%. Histological analysis confirmed tissue structure characteristic of HCC (Fig. 3b). Apoptotic cell death as determined by TUNEL staining was significantly increased in the livers of male Mito-Ob mice compared with control mice (Fig. 3c). A parallel increase in Ki67-positive proliferating cells was also observed in the liver of male Mito-Ob mice (Fig. 3c). Tissue necrosis and anisocytosis of hepatic nuclei were also apparent (Fig. 3b). Thus, male Mito-Ob mice showed continuous hepatocyte death and compensatory proliferation, a critical process in hepatocarcinogenesis^1^,^38^.

### Mito-Ob mice with NASH/HCC exhibit mitochondrial dysregulation

**Figure 4:**
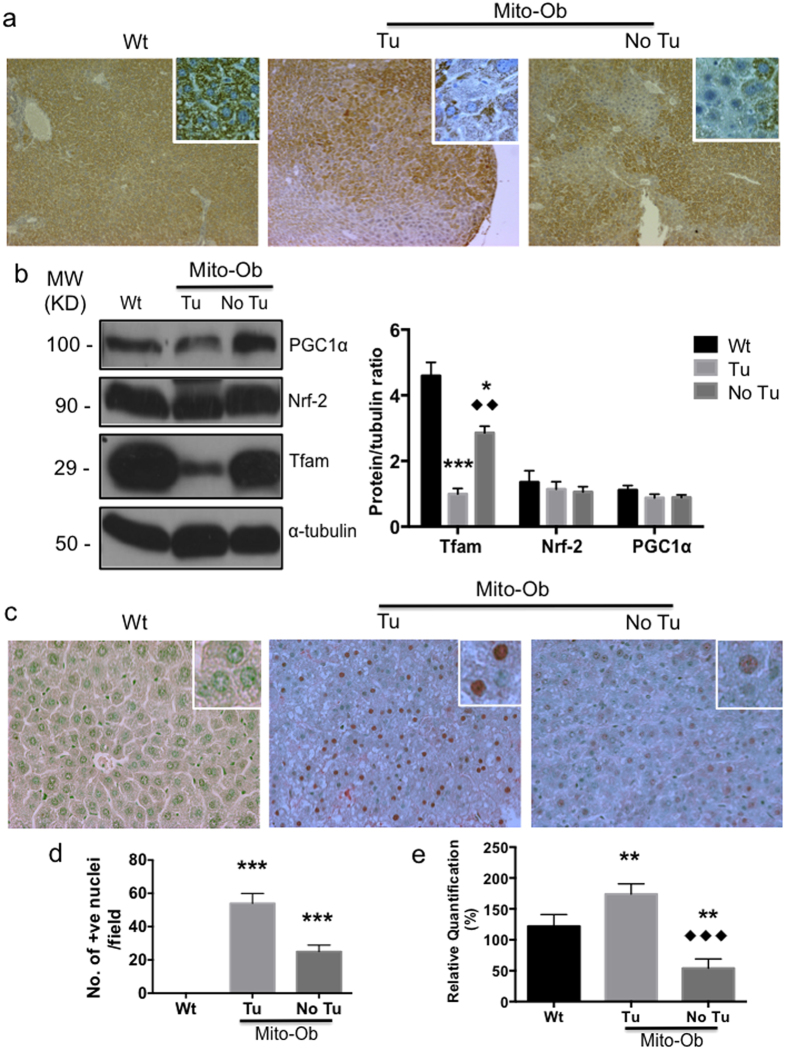
Male Mito-Ob mice with HCC exhibit mitochondrial dysregulation and increased hepatic oxidative DNA damage. (**a**) Representative photomicrographs showing reduction in mitochondria content in the hepatic lesion as determined by immunohistochemistry using anti-prohibitin antibody. Magnification 10X. Magnified view of hepatocytes is shown in the insets. (**b**) Left panel: Representative immunoblots showing expression level of mitochondrial marker proteins in the liver from Mito-Ob mice and control mice. Nrf-2 was used as a control for nuclear transcription factor and beta-tubulin blot is shown as a loading control. Similar conditions were used for the electrophoretic analysis of proteins by Western immunoblotting. Right panel: Histograms showing quantification of protein levels. Data are presented as mean ± SEM (n = 5–7 mice in each group). (**c**) Representative photomicrographs showing oxidative DNA damage in the liver at 12–14 months of age. (**d**) Histograms showing quantification of oxidative DNA damage in the liver as shown in the panel (**c**). (**e**) Histograms showing mitochondrial copy numbers as determined real time-PCR. Data are presented as mean ± SEM (n = 5–7 mice in each group). Asterisks indicate comparison between sex matched Mito-Ob vs Wt. *P < 0.05, **P < 0.01, ***P < 0.001 by Student’s *t* test. Diamonds indicate comparison between Tu and No Tu group by Dunnett’s t test. Wt – wild type; Tu – tumor; No Tu – No tumor; MW – molecular weight; KD – kilo Dalton.

Glucose and lipid toxicity as well as chronic low-grade inflammation are known to cause mitochondrial dysregulation^39^, and emerging evidence suggests that mitochondrial dysregulation in hepatocytes precedes the onset of NASH and HCC^40^. Because the male Mito-Ob mice display all these causative signs, therefore, we sought to know the mitochondrial status in NASH and tumor bearing livers. An immunohistochemical analysis using a mitochondrial marker-specific antibody showed a differential staining pattern in the liver tissue with hepatic lesions, which was significantly diminished in the hepatic lesions compared with the area having normal histological architecture (Fig. 4a), suggesting a relationship between the hepatic lesions and mitochondrial dysregulation. Livers from the wild-type mice showed a homogenous staining pattern (Fig. 4a). The mitochondrial transcription factor A (Tfam) level was significantly decreased in the liver from male Mito-Ob compared with wild type mice as determined by western immunoblotting, which was further reduced in the liver from tumor bearing mice (Fig. 4b). This difference was not observed in the expression level of the nuclear transcription factor-1 (Nrf-2), which is required for nuclear encoded mitochondrial proteins (Fig. 4b). The expression level of PPARγ-coactivator-1α (PGC-1α), showed a trend similar to Tfam protein level, however, it was not statistically different between different groups (Fig. 4b). The mitochondrial DNA copy number was significantly reduced in Mito-Ob mice with NASH development compared with the control liver (Fig. 4e). However in tumor samples, the mitochondrial DNA copy number was up regulated in comparison with control and NASH samples (Fig. 4e).

Taken together, these data suggest that the hepatic phenotype in male Mito-Ob mice mimics obesity-associated mitochondrial dysregulation in steatohepatitis and HCC in humans^40^.

### Mito-Ob mice with HCC showed increased hepatic oxidative DNA damage

To further explore the link between mitochondrial dysregulation and HCC development in male Mito-Ob mice, we measured oxidative DNA damage using the ROS antibody. Hepatic oxidative DNA damage was significantly increased in the liver of tumor bearing Mito-Ob mice compared with Mito-Ob mice without tumors and wild type mice (Fig. 4c,d). An increase in in oxidative DNA damage was also found in Mito-Ob mice without tumors compared to wild type mice; however, it was significantly less than Mito-Ob mice with tumors (Fig. 4c,d). Collectively, these data point towards a role of oxidative stress in the obesity-linked pathogenesis of NASH and HCC.

In this context, it is important to note that mitochondrial impairment and hepatic cell deaths are common features of NASH^15^,^40^, whereas progression from NASH to HCC also involves mark increase in compensatory cell proliferation^1^,^38^. This may be the reason for a negative correlation that was observed between mtDNA copy number and oxidative stress during NASH and HCC development in Mito-Ob mice (Fig. 4c–e). Moreover, this data suggests that an increase in mtDNA may not necessarily mean fully adapted mitochondria.

### Mito-Ob mice with HCC showed reduced liver ghrelin level

**Figure 5:**
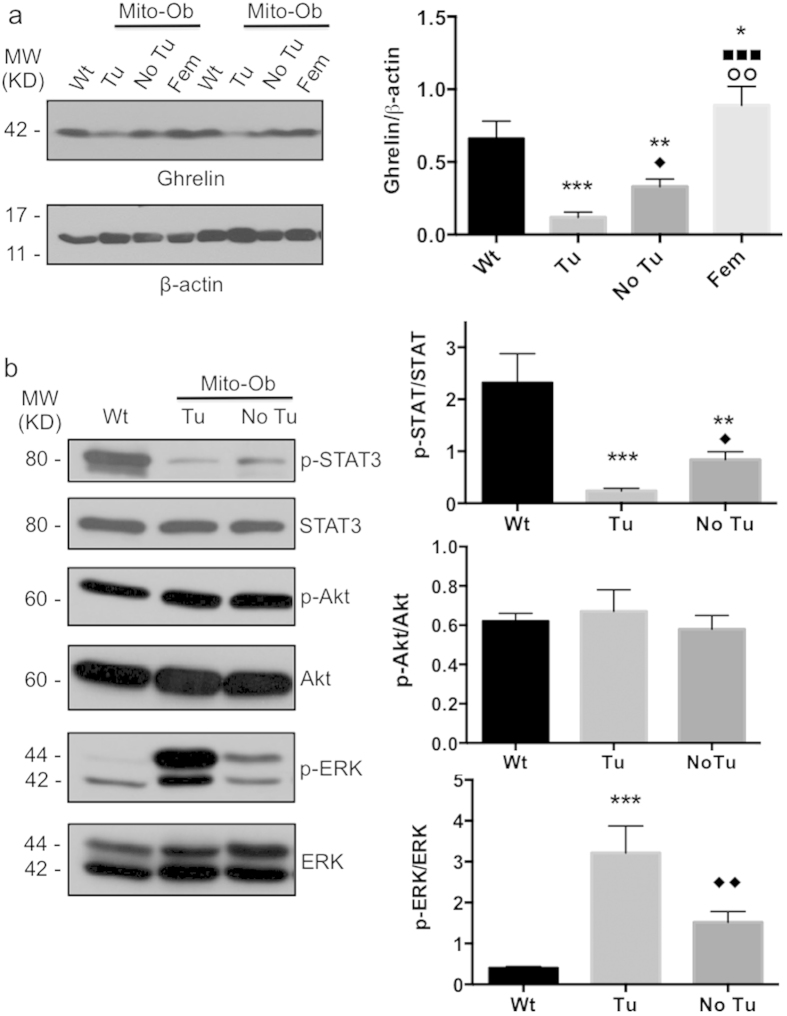
ERK1/2 and STAT3 signaling are inversely altered in the liver tumors from Mito-Ob mice. (**a**) Left panel: Representative immunoblots showing liver ghrelin level at 12–14 months of age as determined by rabbit polyclonal anti-ghrelin antibody. Data from two different animals in each group is shown. Similar conditions were used for the electrophoretic analysis of proteins by Western immunoblotting. Right panel: Histogram showing quantification of the liver ghrelin level as shown in the left panel. Data are presented as mean ± SEM (n = 3–4 mice in each group). Asterisks indicate comparison between sex matched Mito-Ob vs Wt. *P < 0.05, **P < 0.01, ***P < 0.001 by Student’s t test. Diamonds indicate comparison between Tu and No Tu group, square indicate comparison between Tu and Fem and circle indicate comparison between No Tu and Fem by Dunnett’s *t* test. (**b**) Left panel: Representative immunoblots showing the activation level of Akt, ERK, and STAT3 signaling pathways in the liver at 12–14 months of age as determined by their phospho-specific antibodies. Similar conditions were used for the electrophoretic analysis of proteins by Western immunoblotting. Right panel: Histograms showing quantification of the activation level of Akt, ERK, and STAT3 in the liver as shown in the left panel. Data are presented as mean ± SEM (n = 5–7 mice in each group). Asterisks indicate comparison between sex matched Mito-Ob vs Wt. NS, not significant; *P < 0.05, **P < 0.01, ***P < 0.001 by Student’s t test. Diamonds indicate comparison between Tu and No Tu group by Dunnett’s *t* test. ^♦^P < 0.05. Wt – wild type; Tu – tumor; No Tu – No tumor; Fem – female; MW – molecular weight; KD – kilo Dalton.

The pathogenesis of NASH and HCC often associated with increased hepatic inflammation. Because male Mito-Ob mice showed reduced serum ghrelin level and increased hepatic inflammation, therefore, we determined the expression level of ghrelin in the liver from Mito-Ob mice by western immunoblotting. Similar to serum ghrelin levels, tumor bearing liver from male Mito-Ob mice showed significantly reduced ghrelin level in comparison with liver from Mito-Ob mice without tumor and wild type control mice (Fig. 5a). Whereas liver from female Mito-Ob showed significantly increased ghrelin level compared with their male counterparts (Fig. 5a), suggesting a potential role of anti-inflammatory peptide ghrelin in in the development of NASH and HCC in male Mito-Ob mice.

### ERK1/2 and STAT3 signaling are inversely altered in the liver tumors from Mito-Ob mice

To get insight into the cell signaling pathways involved in HCC development in Mito-Ob mice, we determined the activation level of PI3K/Akt, MAPK/ERK and STAT3 signaling pathways because they have been implicated in the pathogenesis of HCC^1^,^26^,^41^, and have been shown to be modulated by PHB in other cell/tissue types^42^. The phopsho-ERK1/2 (p-ERK1/2) level was significantly increased in the liver from male Mito-Ob mice compared with wild type mice, and further increased in the tumor bearing livers (Fig. 5b). In contrast, the phospho-STAT3 (p-STAT3) level showed significant reduction in Mito-Ob mice, and further reduced in tumor bearing mice (Fig. 5b). No significant change in p-Akt levels was found in the liver from Mito-Ob mice with and without tumor in comparison with the wild type mice (Fig. 5b). Taken together, these data suggest a role for increased oncogenic mediator p-ERK and decreased p-STAT3 in the liver tumor development in male Mito-Ob mice.

## Discussion

**Figure 6:**
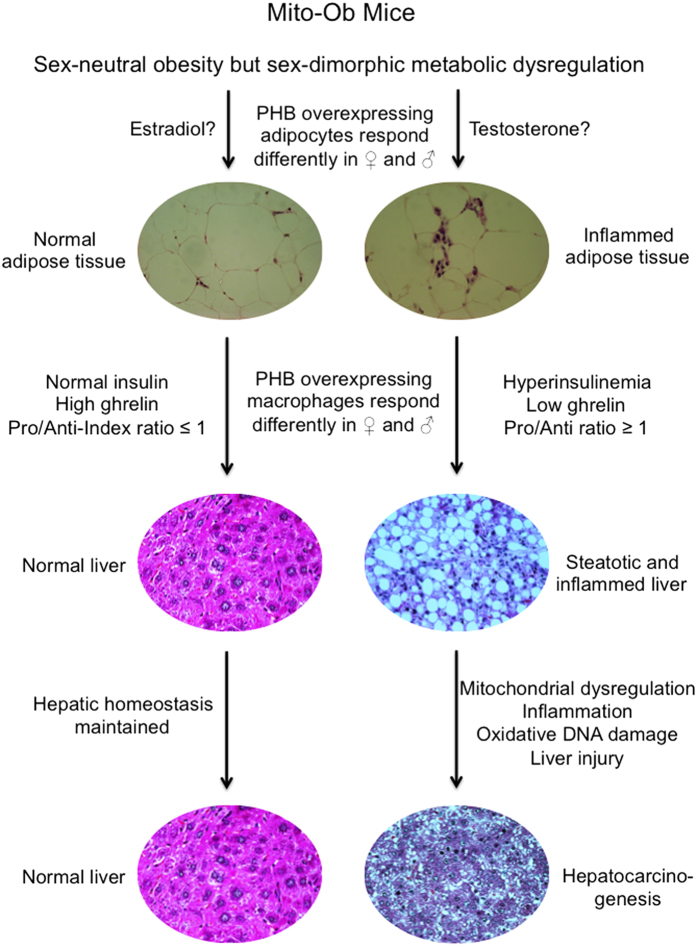
Schematic diagram showing proposed mechanism in obesity-linked NASH and HCC development. We propose that an extensive interplay between the metabolic and inflammatory status at the adipose tissue and systemic levels, as well as at the hepatic tissue level is involved in the progression of obesity-linked hepatic steatosis to NASH and HCC. Pro – proinflammatory; Anti – anti-inflammatory.

This study reports a role of obesity related hyperinsulinemia and chronic low-grade inflammation in the development of NASH and HCC, independent of diet and carcinogen respectively. This pathological progression is marked by adipose inflammation, an inverse alteration of serum insulin and ghrelin levels, an increase in hepatic lipid accumulation, macrophage and lymphocyte infiltration, and a reduction in hepatic mitochondrial content and function, with a parallel increase in hepatic DNA damage, cell death and compensatory proliferation (Fig. 6). Of note, both female and male Mito-Ob mice have a comparable degree of obesity however, only the males displayed pathological features of NASH and HCC. This would suggest that obesity alone is not sufficient for the development of NASH and HCC but rather require additional effects of adipose inflammation, hyperinsulinemia and the degree of hepatic inflammation.

In this context, it is important to note that there are a number of obese rodent models available that display obesity-associated metabolic dysregulation and NAFLD. However, the majority of them do not develop obesity-linked HCC spontaneously like male Mito-Ob mice, except TSOD mice^29^. However, TSOD mice also develop diabetes along with obesity and it remains unclear whether TSOD mice develop HCC due to obesity or diabetes. This raises an important question: *how* and *why* do obesity-associated abnormalities lead to HCC development in male Mito-Ob mice? This may in part be due to a significant reduction in ghrelin levels in male Mito-Ob mice because emerging evidences suggest that ghrelin is an important anti-inflammatory peptide^43^,^44^,^45^,^46^. Since obesity-associated chronic low-grade inflammation in major metabolic tissues is considered an important driver for obesity related disorders, it is possible that the anti-inflammatory function of ghrelin has a role in the sexually dimorphic metabolic phenotype in Mito-Ob mice. A reduced ghrelin level in male Mito-Ob mice may be permissive in the development of obesity-associated chronic low-grade inflammation, which in turn promotes insulin resistance, NASH and HCC. By the same token, an increased ghrelin level in female Mito-Ob mice may have a protective role against obesity-associated low-grade inflammation, and subsequently against insulin resistance and NAFLD.

Furthermore, a parallel decrease in liver ghrelin level in Mito-Ob mice during disease progression as observed in this study may be due to a sex-dimorphic effect of PHB on ghrelin production from macrophages, as macrophages are known to produce ghrelin^47^, which in turn contribute to the development of NASH and HCC in male Mito-Ob mice and confer a protection in female mice. We propose that PHB overexpressing adipocytes and macrophages respond differently in males and females, especially in obesity, and may have a role in sex differences in metabolic dysregulation and adipose inflammation, with as a consequence sex-dimorphic NASH and HCC development in male Mito-Ob mice. It would be interesting to know whether normalization of insulin and/or ghrelin levels would reduce or prevent NASH and HCC occurrence in the male Mito-Ob mice. Of note, insulin sensitizers such as thiazolidinediones (TZDs), glucagon-like peptide-1 receptor agonists and dipeptidyl peptidase 4 inhibitors have been studied as therapeutic approaches for NAFLD in recent years and have been shown to have some beneficial effects^45^. In addition, treatment with anti-inflammatory peptide ghrelin has been reported to reduce inflammation and oxidative stress, NAFLD and NASH^45^,^46^. Thus, a combination therapy of insulin sensitizers with anti-inflammatory agents, especially ghrelin, may provide a better outcome that warrants further investigation. The male Mito-Ob mouse would be a fitting model for such preclinical studies because they display both pathological features: insulin resistance and significantly reduced serum ghrelin levels.

Our data suggest an important role of the oncogenic determinant p-ERK in combination with p-STAT3 down regulation and mitochondrial dysregulation in the development of HCC in male Mito-Ob mice. Whether these changes are related to each other or work independently in HCC development remains to be determined. Glucotoxicity, lipotoxicity, and chronic low-grade inflammation are known to cause mitochondrial dysregulation^39^. A number of studies have reported mitochondrial dysregulation in the liver in the pathogenesis of fatty liver and HCC both in humans and rodents^15^,^16^,^17^,^40^. In addition, recently p-STAT3 has been shown to play an important role in the maintenance of mitochondrial function^48^. A parallel change in mitochondrial dysregulation and the p-STAT3 level during HCC development in male Mito-Ob mice are consistent with the emerging evidence in the literature. However, it is not known whether they coordinate in the development of HCC, which warrants further investigation. In this context, it is important to note that the liver specific Phb knockout (*Phb*−/−) mice have been reported earlier, which also develop HCC by 35–46 weeks of age^49^. HCC development in the *Phb*−/− mice has been attributed to the tumor suppressor function of PHB^49^. However, because PHB is a critical protein for the structural and the functional integrity of mitochondria, and hepatic mitochondrial dysregulation has been associated with HCC development^40^, it is possible that HCC development in the *Phb*−/− mice may in part be due to compromised mitochondrial function. Nevertheless, because PHB has a role as transcriptional co-regulator as well as in membrane signaling^50^,^51^, down regulation of liver PHB expression during HCC development in male Mito-Ob mice, as observed in our data, may also affect its extra-mitochondrial functions along with mitochondrial dysregulation. It is possible that such extra-mitochondrial changes may contribute to HCC development in male Mito-Ob mice.

In summary, our data suggest an extensive interplay between the metabolic and inflammatory status at the adipose tissue and systemic levels, as well as at the hepatic tissue level in the progression of obesity-linked NAFLD to NASH and HCC (Fig. 6). Thus, this study provides a proof-of-concept that obesity-associated metabolic and immune dysregulation indeed have a central role in the pathogenesis of NASH and HCC. In addition, the Mito-Ob mouse revealed a sex-dimorphic role of prohibitin in adipose and immune functions.

## Materials and Methods

### Reagents

Monoclonal anti-PHB antibody was purchased from Cell Signaling Technology (Danvers, MA). Anti-ERK pan, anti-phosphoERK (pERK), anti-Akt pan, anti-phopsho-Akt (pAkt) antibodies were purchased from Cell Signaling Technology (Danvers, MA). Polyclonal anti-ghrelin, anti-mitochondrial transcription factor A (mtTAF), anti-PPARγ-coactivator-1α (PGC-1α) and anti-nuclear respiratory factor-2 (Nrf-2) antibodies were obtained from Abcam Inc. (Toronto, ON). Horseradish peroxidase (HRP)-conjugated secondary antibodies were from Santa Cruz Biotechnology (Santa Cruz, CA) and Cell Signaling Technology (Danvers, MA). All other reagents were purchased from Sigma-Aldrich (Oakville, ON, Canada) unless stated otherwise.

### Transgenic mice

All experiments involving animals were performed as per the study protocols (#12-007/1/2/3) approved by the Animal Care and Use Committee at the University of Manitoba and following the guidelines of the Canadian Council of Animal Care (CCAC). The generation and initial characterization of Mito-Ob mice has been reported in our previous study^35^,^37^. The Mito-Ob transgenic mice were identified by genotyping the tail DNA by PCR using the above forward primer: 5′-GCAGCCCGGGGGATCCACTA-3′ and reverse primer: 5′-GCACACGCTCATCAAAGTCCTCTCCGATGCTG-3′. The animals were given normal chow (LabDiet, St. Louis, MO) and water ad libitum. The body weight and food intake of the Mito-Ob and the wild-type mice were recorded weekly as described previously^35^.

### Histology and immunohistochemistry

Visceral adipose tissue and the liver from 9–12 month old Mito-Ob and their wild type littermates (as applicable) were fixed in buffered formaldehyde (Fisher Scientific, Ottawa, ON) and subsequently dehydrated, embedded in paraffin, and 5 μm sections were stained with hematoxylin-eosin or a protein specific antibody for immunohistochemistry^35^,^37^.

### Western immunoblotting

Liver expression level of mitochondrial proteins including PHB and cell signaling proteins were determined by Western immunoblotting. In brief, the total liver tissue lysates from Mito-Ob and wild-type mice containing equal amount of proteins (~20 μg/lane) were separated by SDS-PAGE and subsequently analyzed by Western immunoblotting using a protein-specific primary antibody and a HRP-conjugated respective secondary antibody as described before^35^,^37^. Finally, immunodetection was performed using an Enhanced Chemiluminiscence kit (GE Healthcare, Mississauga, ON).

### Adipokines and hormones measurements

Adipokines and hormones in the mouse serum were measured using mouse Bio-Plex ProTM Assays Diabetes panel and Bio-Plex 200TM multiplex suspension array systems (Bio-Rad, Hercules, CA) as per the manufacturer’s protocols^35^,^37^.

### Apoptosis, cell proliferation and oxidative DNA damage measurements

Apoptotic cell death using TUNEL assay (Trevigen, Gaithersburg, MD) and cell proliferation using anti-Ki67 antibody (Cell Signaling) were measured following the manufacturer’s protocol described earlier^52^,^53^. Oxidative DNA damage in the liver tissue was measured using a monoclonal antibody from Biorbyt Ltd. (Cambridgeshire, U.K.) and HRP-conjugated secondary antibody and 3,3′-diaminobenzidine enhanced liquid substrate system as substrate (Sigma-Aldrich, Oakville, ON, Canada). This antibody is raised using 8-hydroxy-guanosine-BSA and casein conjugates as an immunogen and has been recommended for human, mouse, and rat tissues as per technical data sheet.

### Mitochondrial DNA (mtDNA)

mtDNA copy number in adipose tissue was determined by real-time PCR^26^ using an Applied Biosystem 7500 machine. Following primers were used for mtDNA and beta-actin control: mtDNA-forward CGACCTCGATGTTGGATCA and mtDNA-reverse AGAGGATTTGAACCTCTGG at annealing temperature 55 °C and beta actin primers: b-actin-forward CCCTACAGTGCTGTGGGTTT and b-actin-reverse GAGACATGCAAGGAGTGCAA at annealing temperature 58 °C^54^.

### Statistical analysis

All statistical analyses were performed using GraphPad Prism 6. Experimental results are shown as mean ± SEM. The two-tailed Student’s unpaired t-test was performed to compare sex-matched Mito-Ob and wild-type littermates. One-way ANOVA with Dunnett’s t-test was performed when comparisons were made between Mito-Ob mice with and without tumors with a control group. Two-way ANOVA was performed with sex and genotype as factors. P-values < 0.05 were considered significant.

## Additional Information

**How to cite this article**: Ande, S. R. *et al*. Prohibitin-induced, obesity-associated insulin resistance and accompanying low-grade inflammation causes NASH and HCC. *Sci. Rep.* **6**, 23608; doi: 10.1038/srep23608 (2016).
